# Molecular detection, seroprevalence, and phylogenetic analysis of *Brucella suis* in wild pigs (*Sus scrofa*) in Iraq: Implications for One Health surveillance

**DOI:** 10.14202/vetworld.2025.2733-2745

**Published:** 2025-09-18

**Authors:** Hasanain A. J. Gharban, Eva Aisser Ajaj, Hadeel Asim Mohammad

**Affiliations:** 1 Department of Internal and Preventive Veterinary Medicine, College of Veterinary Medicine, University of Wasit, Wasit, Iraq; 2 Department of Internal and Preventive Medicine, College of Veterinary Medicine, University of Mosul, Nineveh, Iraq

**Keywords:** *Brucella suis*, Iraq, phylogenetic analysis, quantitative real-time polymerase chain reaction, wild pigs, zoonosis

## Abstract

**Background and Aim:**

Brucellosis remains a globally significant zoonotic disease with significant public health and economic implications. While domestic pigs are absent in Iraq due to religious restrictions, wild pigs (*Sus scrofa*) represent a potential reservoir for zoonotic pathogens, including *Brucella suis*. Despite this, no prior investigations have assessed the prevalence of swine brucellosis in Iraq. This study aimed to (i) determine the seroprevalence of brucellosis in wild pigs using enzyme-linked immunosorbent assay (ELISA), (ii) evaluate associated risk factors such as age, sex, and region, and (iii) confirm infection and characterize isolates through molecular detection and phylogenetic analysis.

**Materials and Methods:**

Between September 2022 and January 2024, venous blood samples (n = 42) from recently killed wild pigs in Wasit province were analyzed. Sera were screened using ELISA, while DNA was extracted and tested with conventional polymerase chain reaction (PCR) and quantitative real-time PCR (qPCR) targeting the 16S ribosomal RNA gene. Positive isolates were sequenced and compared to reference strains in GenBank using phylogenetic analysis. Statistical associations with risk factors were assessed using odds ratios and relative risk.

**Results:**

ELISA detected anti-*Brucella* antibodies in 54.76% of samples, with mild (39.13%), moderate (34.78%), and severe (26.09%) infections. Molecular assays revealed *B. suis* DNA in 33.33% of samples by PCR and 45.24% by qPCR. Seropositivity was significantly higher in pigs aged 2–4 years (73.33%) compared with <2 years (38.1%) and >4 years (66.67%) (p < 0.05). Female pigs exhibited a higher prevalence (57.58%) than males (44.44%), and animals from Al-Numaniyah showed the highest rates (73.68%). Phylogenetic analysis revealed 98.87%–99.76% similarity with Indian *B. suis* strains (MF173089.1), characterized by minor nucleotide variations.

**Conclusion:**

This study provides the first molecular evidence of *B. suis* in wild pigs in Iraq, underscoring their role as potential reservoirs for zoonotic transmission. The findings highlight the importance of integrating molecular diagnostics with serology for accurate surveillance. Strengthening One Health strategies, including wildlife monitoring, biosecurity, and public health education, is critical to preventing spillover to domestic animals and humans. Further large-scale investigations are warranted to better characterize the epidemiology of swine brucellosis in the region.

## INTRODUCTION

Brucellosis is a zoonotic bacterial disease caused by members of the *Brucella genus*. These bacteria are small, non-capsulated, non-motile, facultative, intracellular Gram-negative coccobacilli [1]. A wide variety of domestic animals, wild species, and even marine mammals can become infected with different *Brucella* species [2]. Pigs, both domestic and wild, are considered major reservoirs for several *Brucella* species, particularly *B. suis*, which typically includes three biovars (1, 2, and 3), along with two others (4 and 5) that can be transmitted to pigs from caribou/reindeer and wild rodents, respectively [3, 4]. The primary sources of infection are infected animals and their byproducts (aborted fetuses, placentas, and vaginal discharges), with transmission occurring through both direct and indirect contact [5]. Moreover, the expansion of animal industries and urbanization has been recognized as a significant factor in the spread and persistence of brucellosis [6]. Humans, including veterinarians, farmers, and slaughterhouse workers, are at risk of infection through occupational exposure to contaminated materials [7]. Globally, brucellosis remains a significant health issue, particularly in regions with inadequate sanitation, close contact between humans and animals, and limited access to adequate healthcare [8]. Economically, the disease causes reduced livestock productivity, reproductive losses, and trade barriers, leading to considerable financial damage for farmers and the livestock sector [9].

Accurate laboratory diagnosis of brucellosis remains a challenge [10]. Although bacterial culture is considered the gold standard, it performs poorly, requiring up to 6 weeks and showing variable sensitivity [11]. Therefore, researchers have recommended more reliable diagnostic approaches, such as serological and molecular techniques, to improve detection and guide effective control strategies [12, 13]. Serological tests, ranging from conventional Rose Bengal Test (RBT) to advanced enzyme-linked immunosorbent assay (ELISA), are widely used due to their simplicity and speed [14, 15]. However, their limitations include difficulties in interpreting results caused by endemic titers and non-standardized kits for antibody measurement [14]. To overcome these drawbacks, DNA-based molecular assays such as polymerase chain reaction (PCR) have become the preferred choice, offering rapid detection with high sensitivity and specificity [16]. Furthermore, phylogenetic analysis of bacteria, typically based on specific gene targets, is an essential tool for understanding inter-species or intra-species relationships, characterizing new pathogens, and supporting the development of novel therapeutic strategies [17, 18].

Wild pigs (*Sus scrofa*), also referred to as feral hogs or boars, are increasingly recognized as important reservoirs for a range of pathogens. These animals can carry and transmit diseases that affect domestic livestock, wildlife, and humans, including bacterial infections (leptospirosis, brucellosis, salmonellosis), parasitic diseases (trichinellosis, toxoplasmosis), and viral infections (hepatitis E, swine influenza) [19, 20]. In recent decades, wild pig populations have expanded rapidly due to habitat loss, absence of natural predators, and human-related factors such as farming practices and recreational hunting. As they invade new territories, their interactions with domestic animals increase the risk of cross-species disease transmission [21, 22].

Despite brucellosis being one of the most studied zoonoses worldwide, majority of the investigations have focused on *Brucella* abortus and *Brucella* *melitensis* in ruminants, given their well-recognized impact on livestock productivity and public health. In contrast, *Brucella* suis, particularly in wild pigs (*S. scrofa*), remains underexplored in many regions, including the Middle East. While domestic pigs are well-established reservoirs of *B. suis* in several countries, Iraq represents a unique case where religious restrictions preclude pig farming, thereby creating a research vacuum on porcine brucellosis. Nonetheless, wild pig populations are present across Iraqi agricultural landscapes, where they frequently interact with domestic livestock, farmers, and shared environments. This ecological interface creates a potential risk of *Brucella* transmission that has not yet been quantified.

Furthermore, no published reports have addressed the seroprevalence, molecular detection, or phylogenetic characterization of *B. suis* in Iraq. Previous studies in the country have focused solely on other *Brucella* species in cattle, buffaloes, sheep, goats, camels, and dogs, leaving the epidemiological role of wild pigs entirely neglected. Without such data, the possible contribution of wild pigs to the maintenance and spread of brucellosis in Iraq remains unknown. Moreover, while advanced molecular tools such as qPCR and phylogenetic sequencing have been widely applied in global investigations, their use in Iraqi studies of wildlife-associated brucellosis has been almost non-existent. This gap limits both the national understanding of *B. suis* circulation and the development of evidence-based One Health strategies for controlloing zoonotic disease.

The present study was designed to address these knowledge gaps by providing the first systematic investigation of swine brucellosis in Iraq. Specifically, it aimed to:

1. Determine the seroprevalence of brucellosis in wild pigs using enzyme-linked immunosorbent assay (ELISA)
2. Identify epidemiological risk factors (age, sex, and geographic location) associated with seropositivity
3. Confirm infection and assess prevalence using molecular approaches, including conventional PCR and quantitative real-time PCR (qPCR)
4. Characterize local *B. suis* isolates phylogenetically through sequencing and comparison with international reference strains.

By integrating serological and molecular tools, this study provides novel insights into the presence and genetic relatedness of *B. suis* in Iraqi wild pigs. The findings are expected to contribute to a more comprehensive understanding of the epidemiology of brucellosis in Iraq, highlight the importance of wildlife surveillance, and support the development of integrated One Health measures to prevent potential spillover into domestic livestock and humans.

## MATERIALS AND METHODS

### Ethical approval

The current study was approved by the Scientific Committees of the College of Veterinary Medicine at the University of Wasit and the College of Veterinary Medicine at the University of Mosul (Ref. No. UM.VET.2025.009 in 15-01-2025).

### Study period and location

The study was conducted from September 2022 and January 2024. The study animals were selected from various agricultural, rural, and suburban areas in Wasit Province, which is situated in the southeastern part of Iraq, bordering Iran to the east and located at geographical coordinates of 32°00′–33°30′ N and 44°30′–46°40′ E. The capital city, Al-Kut, is located about 176 km south of Baghdad province (the capital). All areas in Wasit province experience a transitional climate that blends Mediterranean and desert elements. Summers are hot and dry, with extremely high temperatures, while winters are mild, offering cooler conditions compared to the summer months. The study areas are largely flat and plain and include parts of the Mesopotamian Marshes, specifically the Shuwayja, Al-Attariyah, and Hor Aldelmj marshes. The terrain is predominantly alluvial, characterized by fertile soil that is well-suited for agriculture, particularly in areas near rivers and marshes. The proximity to the Tigris River and its tributaries significantly contributes to the region’s agricultural potential.

### Study animals

A total of 42 recently killed wild pigs (within 3 h) were randomly selected from various agricultural, rural, and suburban areas in Wasit province. The selection of study animals was not based on a specific formula, but rather followed the farmers’ calls. In addition, blood samples were not obtained from many killed wild pigs because the farmers discarded or destroyed the carcasses of the animals.

### Collection of samples and data

Approximately 5 mL of jugular venous blood was collected from each study animal using a disposable syringe. Each sample was divided into a 2.5 mL ethylenediaminetetraacetic acid (EDTA)-anticoagulant plastic tube that was frozen at –20°C until molecular testing, and the remaining blood was transferred into a glass-gel tube that was centrifuged(4,193 × *g*, 5 min). The obtained sera were transferred into labeled Eppendorf tubes and kept frozen until serological testing. The age, region, and sex of the study animals were recorded as risk factors. The age of the study animals was determined based on tooth eruption and estimated wear.

### Serological examination using ELISA

According to the protocol described in the Anti-*Brucella* ELISA Kit ([Abbexa, UK], Catalog No.: abx365011), the serum samples and kit components were prepared and processed, and the ODs were read using the automated microplate photometer (BioTek, USA) at a wavelength of 450 nm. To estimate the seropositive samples, the value of the blank was set to zero, and the percentage inhibition (PI) value was calculated according to the following formula:

PI = PI = ([1 − OD_Sample_]/OD_Negative Control_) × 100

The test was validated based on the results of the positive and negative controls, and the results of the study samples were interpreted as positive if PI > 70% and negative if PI ≤ 70%. The severity values among the seropositive ODs were further classified into mild (PI = 70%–<75%), moderate (PI = 75%–<80%), and severe (PI > 80%) infections.

### DNA extraction and quality control

After thawing the EDTA-blood samples, the manufacturer’s instructions of the Type A Protocol in the G-Spin Total DNA Extraction Kit (iNtRON Biotechnology, South Korea) were followed to extract DNA. The extracted DNA samples were then examined using the Nanodrop Spectrophotometer System (Thermo Scientific, UK) to evaluate their concentration (ng/μL) and purity at an absorbance ratio of A260/A280 nm.

### Conventional PCR assay

Based on the NCBI-GenBank *B. suis* isolate (ID: L26169.1), one set of primers (F [5´-AAG GCG ACG ATC CAT AGC TG-3´] and R [5´-TAT CAC CGG CAG TCC CCT TA-3´]) was designed to prepare the MasterMix tubes at a final volume of 20 μL using the AccuPower PCR PreMix Kit (Bioneer, South Korea). In the Thermal Cycler System T100 (Bio-Rad, USA), the PCR reaction was optimized as follows: 1 cycle initial denaturation (95°C/5 min), 30 cycles of denaturation (95°C/30 s), annealing (58°C/30 s), extension (72°C/30 s), and 1 cycle final extension (72°C/7 min). Electrophoresis for PCR products was performed in a 1.5% agarose gel stained with ethidium bromide at 100 V and 80 mA for 90 min, after which the positive samples were observed under a ultra-violet illuminator (Clinx Science, China) at approximately 874 bp [23].

For the molecular assay, the number of tested samples at each time point was unequal, as it depended on the availability of newly obtained samples to avoid DNA damage. Moreover, agarose gel electrophoresis was performed initially to detect suspected and definite positive samples, and later, all these initially tested samples were retested for confirmation. No positive control was applied in each DNA amplification, and only distilled water was used as a negative control.

### Quantitative real-time PCR (qPCR) analysis

Following the manufacturer’s instructions for the GoTaq qPCR Master Mix Kit (Promega, Korea), the extracted DNA samples from the conventional PCR assay, together with one set of primers (F [5´-AGT TTA CTC CTT CGG TGG CG-3´] and R [5´-AAG GGC TGG TAA GGT TCT GC-3´]) designed in the current study based on the NCBI-GenBank *B. suis* isolate (ID: KY576800.1), were used to prepare the MasterMix tubes at a final volume of 25 μL. For the PCR reaction, the MasterMix tubes were subjected to the Real-Time PCR System (Bio-Rad, USA) under the following conditions: one cycle of pre-denaturation (95°C/5 min), followed by 40 cycles of denaturation (95°C/20 s) and annealing (55°C/1 min), with a melting temperature range of 65°C–90°C. The expected product size was 154 bp. qPCR data were analyzed by calculating the threshold cycle number (CT value), positive amplification, and negative control sample [24]. No positive control was used in the molecular examination because neither previous studies nor specific centers in Iraq could provide such materials. Distilled water was used as a negative control for each group of tested samples.

### Sequencing and phylogenetic analysis

To register the local *B. suis* isolates in the National Center for Biotechnology Information database, DNA from five positive samples identified by conventional PCR was sequenced using the Sanger method at Macrogen Company (South Korea). The obtained sequences were submitted to NCBI-GenBank and assigned specific accession numbers (PP564765.1, PP564766.1, PP564767.1, PP564768.1, and PP564769.1). They were then subjected to Clustal W alignment and Maximum Likelihood analysis in MEGA-11 software for phylogenetic tree construction and homology sequence identity assessment to detect similarities and mutations/changes between the local isolates and *B. suis* or other *Brucella* species isolates from NCBI-BLAST.

### Statistical analysis

All study data were analyzed using t-tests and one-way analysis of variance in GraphPad Prism Software version 8.0.2 (GraphPad Software Inc., USA). Odds ratios and relative risks were calculated online using MedCalc Statistical Software version 22.00.1 (MedCalc Software Ltd, Belgium) based on the number of exposed animals (positive) in each group. Values were represented either as mean ± standard error or as number (percentage) (No. [%]). Variation between study values was considered statistically significant at p < 0.05 (*), p < 0.01 (**), p < 0.001 (***), and p < 0.0001 (****), with results also presented with 95% confidence intervals (95% CI) [25].

## RESULTS

### Serological findings

**Figure 1:**
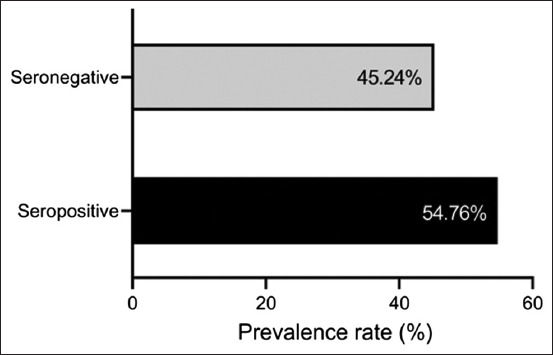
Serological results of enzyme-linked immunosorbent assay for testing the sera of wild pigs (n = 42).

**Figure 2:**
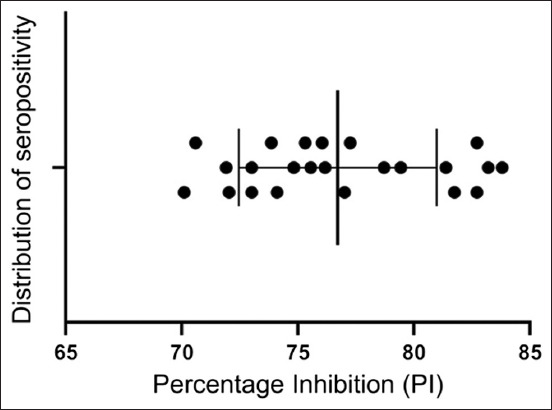
Distribution of seropositive percentage inhibition values among infected wild pigs (n = 23).

Serological examination of 42 sera by ELISA showed that 54.76% (n = 23) of wild pigs were positive for anti-*Brucella* antibodies, which were distributed as mild (39.13%), moderate (34.78%), and severe (26.09%) infections (Figure 1 and Table 1). Subsequently, the positive ELISA findings revealed the presence of significant variation in PI values among the mild (72.61 ± 0.53), moderate (76.94 ± 0.52), and severe (82.60 ± 0.37) infections (p < 0.0001; 95% CI: 74.88–78.56) (Figure 2 and Table 1).

**Table 1:** Classification of seropositive results according to infection severity.

Infection	No. (%)	PI value (M ± SE)
Mild	9 (39.13)	72.61 ± 0.53
Moderate	8 (34.78)	76.94 ± 0.523
Severe	6 (26.09)	82.60 ± 0.368
p-value	0.013*	0.0014**
95% CI	16.84 to 49.83	64.94–89.83

PI = Percentage inhibition, CI = Confidence interval, M = Mean, SD = Standard error, Significance:

* (p < 0.05),

** (p < 0.01).

### Molecular detection by PCR and qPCR

**Figure 3:**
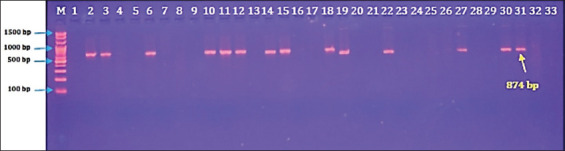
Agarose gel electrophoresis of polymerase chain reaction products at 100 V and 80 mA for 90 min. Lane (M): ladder marker (100 bp–1500 bp). Lanes (1, 4, 5, 7, 8, 9, 16, 17, 20, 21, 23, 24, 25, 26, 28, 29, 32, and 33): Negative samples. Lanes (2, 3, 6, 10, 11, 12, 14, 15, 18, 19, 22, 27, 30, and 31: Positive samples at ≈ 874 bp.

**Figure 4:**
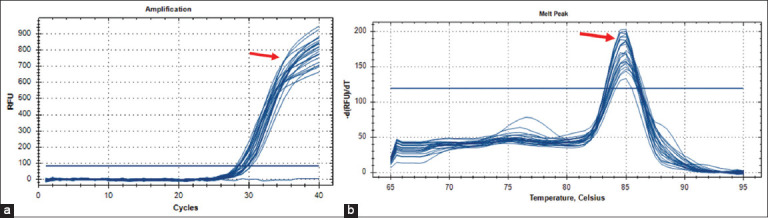
Quantitative polymerase chain reaction results for *the 16S ribosomal* *RNA* gene in *Brucella suis*-positive samples; (a) amplification plot (b) melting peak.

Targeting the 16S ribosomal RNA gene, qualitative PCR testing of 42 DNA samples revealed that 33.33% (n = 14) of wild pigs were positive for *B. suis* infection (Figure 3). Quantitatively, the overall results of qPCR showed that 45.24% (n = 19) of wild pigs were positive for *B. suis* infection (Figure 4a). In addition, amplification of DNA using fluorescent molecules revealed that the cycle threshold (Cq) values of positive samples ranged between 26–28 cycles (Figure 4b).

### Association with risk factors

A significant variation (p < 0.05) was detected in the distribution of seropositive results among the assessed risk factors (Table 2). Regarding age, the highest rate and risk of brucellosis were observed in pigs aged 2–4 years (73.33%, 1.3750, and 3.4375, respectively), followed by those aged >4 years (66.67%, 1.1579, and 1.7895, respectively). In contrast, pigs younger than 2 years exhibited a significantly reduced prevalence (38.1%, 0.6621, and 0.2462, respectively). The prevalence and risk of brucellosis were also significantly higher in Al-Numaniyah (73.68%, 1.5084, and 4.3556, respectively), followed by Al-Kut (58.33%, 1.0592, and 1.2250, respectively) and Al-Swairah (20%, 0.4206, and 0.1310, respectively), while no positive results were recorded in Al-Hai (0%). With respect to sex, female pigs showed a higher seropositivity rate and risk (57.58%, 1.1875, and 1.6964, respectively) compared to males (44.44%, 0.8421, and 0.5895, respectively).

**Table 2:** Association of seropositivity (total number = 23) with risk factors (age, region, and sex).

Factor (group)	Positive/total no.	Prevalence	Risk	Odds ratio
Age (Year)				
<2	8/21	38.1%	0.6621	0.2462
2–4	11/15	73.33%	1.3750	3.4375
>4	4/6	66.67%	1.1579	1.7895
p-value		0.0316*	0.0001****	0.0001****
95% CI		12.87–105.9	15.73–197.3	21.40–578.9
Region				
Al-Swairah	2/10	20%	0.4206	0.1310
Al-Numaniyah	14/19	73.68%	1.5084	4.3556
Al-Kut	7/12	58.33%	1.0592	1.2250
Al-Hai	0/1	0	0	0
p-value		0.0111*	0.0001****	0.0001****
95% CI		15.99–92.00	0.3171–18.11	1.798–46.54
Sex				
Male	4/9	44.44%	0.8421	0.5895
Female	19/33	57.58%	1.1875	1.6964
p-value		0.0295*	0.0005***	0.0001****
95% CI		32.47–134.5	1.180–32.09	5.889–81.75

CI = Confidence interval, Significance:

* (p < 0.05),

**(p < 0.01),

*** (p < 0.001), and

**** (p < 0.0001).

### Phylogenetic analysis

**Figure 5:**
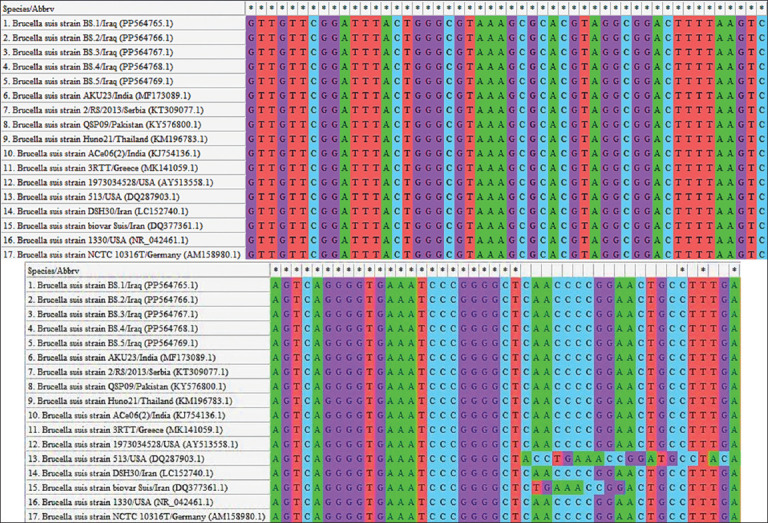
Multiple sequence alignment of local and National Center for Biotechnology Information-GenBank *Brucella suis* isolates.

**Figure 6:**
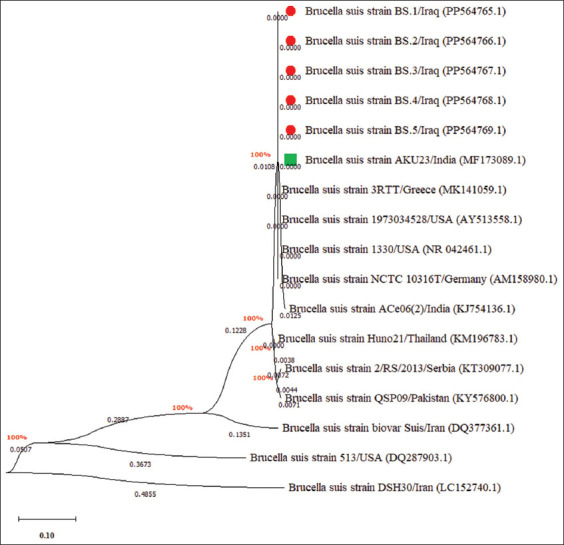
Phylogenetic tree analysis of local and National Center for Biotechnology Information-BLAST *Brucella suis* isolates.

Phylogenetic analysis revealed that the local *B. suis* isolates were closely related to the NCBI-BLAST *B. suis* Indian strain (ID: MF173089.1), with a total genetic similarity ranging from 98.87% to 99.76% and mutation/changes of 0.0024–0.0134% (Figures 5 and 6 and Table 3).

**Table 3:** Homology sequence identity for local *B. suis* and NCBI-BLAST isolates.

Local isolate		NCBI isolate			
	
Name	Accession No.	Species	Country	Accession no.	Identity (%)
BS.1	PP564765.1	*B. suis*	India	MF173089.1	99.70
BS.2	PP564766.1	*B. suis*	India	MF173089.1	99.65
BS.3	PP564767.1	*B. suis*	India	MF173089.1	98.87
BS.4	PP564768.1	*B. suis*	India	MF173089.1	99.76
BS.5	PP564769.1	*B. suis*	India	MF173089.1	98.89

*B. suis = Brucella suis*, NCBI = National Center for Biotechnology Information

## DISCUSSION

### Wild pigs as reservoirs of zoonotic pathogens

Worldwide, wild pigs act as reservoirs and amplification hosts for numerous zoonotic and transboundary pathogens, posing considerable public and animal health challenges [26, 27]. The inherent genetic and physiological similarities between pigs and humans, coupled with intensive farming practices, create an ideal environment for pathogen evolution and cross-species transmission, which is often exacerbated by the robust immune responses of pigs that can mask infection while promoting shedding [28–30]. The continuous co-circulation of various endemic pathogens, including bacterial (leptospirosis, brucellosis, and salmonellosis), parasitic (trichinellosis and toxoplasmosis), and viral (hepatitis E and swine influenza), often leads to complex polymicrobial infections in swine, further complicating control efforts [31, 32]. This intricate web of factors underscores the urgent need for enhanced surveillance measures to mitigate the risks posed by swine-borne pathogens to global health and economic stability [33, 34].

### Brucellosis studies in Iraq and global comparisons

Several studies have been conducted in Iraq to detect various *Brucella* species (*B. abortus, B. melitensis*, and *Brucella ovis*) in different animals, including cattle, buffaloes, sheep, goats, camels, horses, and dogs [15, 35–47]. However, due to religious restrictions, no studies have been conducted in Iraq. In this study, the prevalence rates of brucellosis in wild pigs using three diagnostic tools, namely ELISA, PCR, and qPCR, were 54.76%, 33.33%, and 45.24%, respectively.

Globally, different prevalence rates have been reported. Akoko *et al*. [48] reported an incidence rate of *Brucella spp*. in Kenya of 0.57% in slaughtered domesticated pigs, determined by both serological and molecular assays, with *B. abortus* being the most prevalent species. In Pakistan, the seroprevalence of swine brucellosis was 16.7% by RBT, 12.5% by ELISA, and 23.53% by PCR [49]. A comprehensive literature review by Gong *et al*. [50] revealed that the prevalence varies significantly by region (Africa [1.7%], America [16.5%], Asia [0.5%], Europe [17.4%], Oceania [0.6%]), sampling years (2000 or before [0.7%], 2001–2005 [0.7%], 2006–2010 [2.7%], 2011–2015 [0.6%], 2016 or later [0.7%]), income level (low [0.1%], middle [0.8%], high [15.7%]), detection method (Complement fixation test [26.5%], ELISA [9.4%], PCR [16.3%], RBT [1.4%], tube-agglutination [0.5%], others [11%]), season (spring [2.6%], summer [11.1%], autumn [0.7%], winter [1.2%]), gender (males [7.9%], females [5.1%]), pig age (adult [4.9%], young [2.1%], weaning [1%]), feeding mode (extensive [2.5%], intensive [0.5%]), and classification (domestic [1.1%), feral [15%]).

Other reports include: Elmonir *et al*. [51] in Egypt, who recovered 1.3% of *Brucella* isolates from 1116 slaughtered domestic pigs confirmed as *B. suis*; Zhang *et al*. [52] in China, who found a seroprevalence of 0.11% among 2816 slaughtered pigs; PCR detection of *B. suis* in 26.3% of Spanish environmental samples [53]; a European study [54] have reported seropositivity in 23.02% of domestic pigs and 36.73% of wild pigs with molecular confirmation in 89.66% and 83.33%, respectively; and in India, a seroprevalence of 5.25% by RBT and 10.14% by ELISA, with 9.1% confirmed by both ELISA and PCR [55].

### Diagnostic approaches and their limitations

In this study, the serological findings and both qualitative and quantitative diagnostics showed significant variation in their positive values. Accurate and timely diagnosis of infectious diseases is crucial for effective treatment and disease management. Although traditional approaches such as serology and culture-based methods have been widely used, the emergence of molecular diagnostic techniques has significantly improved the accuracy and speed of pathogen detection. However, discrepancies have been observed between serological and molecular tests in clinical settings, creating challenges for healthcare professionals [56–58]. Diagnostic accuracy, sensitivity, and specificity are essential considerations when comparing serological with molecular methodologies [59].

Molecular methods, such as PCR, provide high sensitivity, can distinguish between active and recent infections, and amplify DNA from pathogen genetic material [60, 61]. In contrast, serological techniques may poorly detect recent infections but can rapidly identify previously infected individuals, including asymptomatic cases, making them useful in surveillance and outbreak investigations [15, 16, 62].

### Fundamental differences between serological and molecular tests

One of the main reasons for discrepancies between serological and molecular diagnostics lies in their underlying principles. Serological tests detect antibodies produced by the host’s immune response, providing evidence of past exposure but not always indicating active infection [25, 56, 63]. Molecular diagnostics, on the other hand, directly identify pathogen-specific DNA, allowing detection even in the absence of an antibody response. This is particularly important for recent infections or immunocompromised individuals with weak antibody production [23, 64]. In addition, molecular methods can identify non-culturable pathogens such as viruses and fastidious bacteria, which are difficult to detect using culture-based methods [65–67].

### Risk factors: Age, region, and sex

In this study, analysis of risk factors revealed significantly higher brucellosis prevalence in pigs aged 2–4 years compared with those <2 years or >4 years. These results align with Sapundzic *et al*. [68], who reported higher susceptibility in wild pigs aged 1.5–2.5 years (51.11%) and 6–18 months (31.11%) compared to younger (0–6 months, 6.67%) and older (>2.5 years, 11.11%) groups. This may reflect increased neonatal mortality in piglets from *B. suis*-infected sows or the influence of maturity and mating on infection transmission. Similarly, Olsen and Tatum [69] found that most pigs infected in utero cleared *B. suis* by 6 months, though a small percentage remained positive beyond 2 years of age.

Regional variations showed higher seropositivity in Al-Numaniyah than in other locations, which may partly reflect the larger number of animals tested there. Environmental and management factors may also support prolonged survival of *B. suis* and increased brucellosis incidence.

Sex-related differences indicated that females exhibited higher seropositivity than males, consistent with some studies [70, 71], although contrasting with others that reported no difference or higher susceptibility in males [50, 51, 68, 72, 73]. This finding could be due to the larger number of tested females or the role of the female reproductive tract as a reservoir for pathogen persistence.

## CONCLUSION

This study provides the first molecular evidence of *B. suis* infection in wild pigs (*S. scrofa*) in Iraq, highlighting their potential role as reservoirs of zoonotic pathogens within the One Health interface. Using a combination of serological and molecular tools, we found that 54.76% of animals tested positive for anti-*Brucella* antibodies by ELISA, with infection severity distributed as mild (39.13%), moderate (34.78%), and severe (26.09%). Molecular detection confirmed infection in 33.33% of animals using conventional PCR and 45.24% using qPCR, with positive amplification cycles ranging from 26 to 28. Risk factor analysis revealed a significantly higher prevalence in pigs aged 2–4 years, in females, and in animals from Al-Numaniyah, suggesting epidemiological hotspots and population groups that require targeted monitoring. Phylogenetic analysis revealed a high degree of genetic similarity (98.87%–99.76%) between Iraqi *B. suis* isolates and Indian reference strains, characterized by only minor nucleotide variations.

These findings underscore the importance of including wild pigs in national brucellosis surveillance systems. Early detection through ELISA and molecular assays offers valuable tools for monitoring potential spillover to livestock and humans. The strong genetic relatedness of Iraqi isolates with international strains emphasizes the transboundary risk of *B. suis* and the need for regionally coordinated control strategies.

The study’s strengths lie in the integration of serological and molecular approaches, which provide complementary insights into both past exposure and active infection, as well as the inclusion of phylogenetic analysis that places Iraqi isolates into a global context. However, limitations included the small sample size due to difficulties in sample acquisition, lack of cooperation from some farmers, extended collection intervals, absence of positive molecular controls within Iraq, and reliance on expensive diagnostic kits without external funding. These factors may have limited the broader generalizability of the results.

Future work should expand surveillance with larger sample sizes across multiple provinces, combined with environmental sampling and whole-genome sequencing to refine the epidemiological picture. Integrating serological screening of at-risk human populations such as farmers, hunters, and veterinarians could strengthen One Health interventions. Meanwhile, ecological studies assessing wild pig movement patterns would support targeted disease control measures.

In conclusion, wild pigs in Iraq represent an overlooked but significant reservoir of *B. suis*, with clear implications for animal and human health. This study emphasizes the importance of adopting integrated One Health approaches, which combine wildlife surveillance, livestock biosecurity, and public health education, to effectively mitigate the risks of brucellosis transmission. Strengthening intersectoral collaboration and investment in molecular diagnostics will be key to controlling this persistent zoonotic threat.

## DATA AVAILABILITY

All generated data are included in the manuscript.

## AUTHORS’ CONTRIBUTIONS

HAJG: Collection of blood samples, qualitative molecular examination, and phylogenetic analysis of local isolates. EAA: Quantitative molecular examination of study samples. HAM: Serological examination and statistical analysis of the obtained results. HAJG, EAA, and HAM: Designed the study and drafted and revised the manuscript. All authors have read and approved the final manuscript.
